# Supplementary material for: Impact of the implementation of Identification-Situation-Background-Assessment-Recommendation (ISBAR) tool to improve quality and safety measure in a lithotripsy and endourological unit
Source: PLoS One. 2023 Jun 2;18(6):e0286565. doi: 10.1371/journal.pone.0286565 (PMC10237371; doi:10.1371/journal.pone.0286565)
Supplement: Attachment — Submitted filename: Response to Reviewers.docx [file pone.0286565.s001.docx]

Valencia, 11th May 2023

Dear editor,

First, we would like to thank you once again for the time you have taken to review our manuscript entitled “Impact of the implementation of Identification-Situation-Background-Assesment-Recommendation (ISBAR) tool to improve quality and safety measure in a lithotripsy and endourological Unit”. PONE-D-22-33805

We have made a number of changes to the manuscript, according to the editor’s and reviewers’ comments and suggestions. We have now provided a detailed list of these changes. In the manuscript, we have made different changes, which are marked in yellow. Your comments were very useful and have allowed us to improve our work to make it more interesting, attractive, and useful for the scientific community.

Finally, we would like to thank you once again for your work, as your contributions have allowed us to improve this work and thus improve existing knowledge in the analysis of quality in initial pediatric trauma care. We hope that the revised manuscript will be accepted for publication in the Nursing in Critical Care Journal.

Sincerely,

The authors

| COMMENTARIES TO EDITOR | |
| --- | --- |
| Editor Comments to the Author: | |
| 1. Please ensure that your manuscript meets PLOS ONE's style requirements, including those for file naming. 2. Please clarify what you mean by prospective collecting data and add the according details in your revised manuscript." 3. Please ensure that you have specified (1) whether consent was informed and (2) what type you obtained (for instance, written or verbal, and if verbal, how it was documented and witnessed). If your study included minors, state whether you obtained consent from parents or guardians. If the need for consent was waived by the ethics committee, please include this information. 4. We note that you have stated that you will provide repository information for your data at acceptance. Should your manuscript be accepted for publication, we will hold it until you provide the relevant accession numbers or DOIs necessary to access your data. If you wish to make changes to your Data Availability statement, please describe these changes in your cover letter and we will update your Data Availability statement to reflect the information you provide. 5. Please include a caption for figure 1. 6. Please review your reference list to ensure that it is complete and correct. If you have cited papers that have been retracted, please include the rationale for doing so in the manuscript text, or remove these references and replace them with relevant current references. Any changes to the reference list should be mentioned in the rebuttal letter that accompanies your revised manuscript. If you need to cite a retracted article, indicate the article’s retracted status in the References list and also include a citation and full reference for the retraction notice. | 1. We have revised the style requirements, it has been changed and marked in yellow. Thank you for your helpful feedback. 2. We have revised this paragraph and added the information you reclaimed. Thank you for your comment (6^th^ Page). 3. We have rewritten the i paragraph on informed consent to clarify it. Thank you for this correction, which helped us improve the manuscript (8^th^ Page). 4. There is no wish to describe these changes. Thank you. 5. It is included in Page 8. Thank you for your help. 6. It has been revised and modified. Thank you for your feedback. |
| Reviewer 1 | |
| 1.1 I read with great interest the manuscript by Gadea-Company et al. on the implementation of ISBAR tool to improve quality and safety measures. The paper is relevant and sound. However, I have some minor issues to be addressed. 1.2 Title. Please modify into "IMPACT OF THE IMPLEMENTATION OF IDENTIFICATION-SITUATION-BACKGROUND-ASSESSMENT-RECOMMENDATION (ISBAR) TOOL TO IMPROVE QUALITY AND SAFETY MEASURE IN A LITHOTRIPSY AND ENDOUROLOGICAL UNIT"  1.3 Introduction. - Acronyms should be written in full the first time they appear in text. Please modify into: "The mnemonic tool Identification-Situation-Background-Assessment-Recommendation (ISBAR) was established to improve..." - You should mention the surgical technique in use for percutaneous nephrolithotomy (doi: 10.1007/s00345-017-2001-0) and state that it could be performed under general (doi: 10.4111/kju.2013.54.3.172) and spinal anesthesia (doi: 10.23736/S0375-9393.22.16969-5). Please briefly discuss and add these 3 references.  1.4 Results. - Please specify the acronym NPR in Table 5.  1.5 Discussion. - Simulation for surgical training is also a valid and well known tool to increase patient safety and quality of surgical procedures (doi: 10.1016/j.nec.2014.11.002 - doi: 10.1136/qshc.2010.042424). Please discuss. | 1.1Thank you for your kind words.  1.2Title has been modified as you suggested.  1.3It has been modified has you suggested.  It has been discuss briefly surgery has you suggested. Thank you.  1.4 The acronym NPR has been explained (page 14)  1.5We have not talk about simulation for surgical training because it is another article we are working on that will talk about simulation and ISBAR. In this article we talk about simulation as they need to go throw the tool several times. |
| Reviewer 2 | |
| 1. 2. 2.1. Aim of this manuscript is to assess the impact of introduction of surgical safety checklist on perioperative outcomes of endourology procedures in a large referral center. Topic is relevant and efforts of Authors are praiseworthy; however, the manuscript shows several inherent flaws. -Firstly, the retrospective nature of the study impacts on the overall reliability of results. Moreover, the Authors only included patients treated with "high-risk" endoscopic procedures (PCNL) due to the increase likelihood of periop complications and also they excluded patients with high surgical risks. These arbitrary selections negatively impact on the reliability of results. -The Authors concluded that the introduction of surgical safety checklist reduced rate of sepsis and that also influenced lenght of hospital stay. However, difference in terms of sepsis rates between the two study groups was not statistically significant. Therefore, these conclusions cannot be drawn. | 1. 2.   Thank for your words and your time to suggests improvements to our work. The inclusion criteria was thought calmly. We choose PCNL because was the procedure with higher probability of surgical and postoperative complications. The other patients according to our unit results in NPR and analysis of modal effects (from studies that the unit did before) doesn’t shown so many complications as the PCNL had.  On the other hand, there wasn’t a surgical safety checklist as we know, there is a tool that provides the team a good communication to do not cause adverse events and it has demonstrated that using ISBAR there was less complications than it was before its implementation. |
| Reviewer 3 | |
| 1-Why do you compared two non-equal numbers of patients? 2-As long as it is in urological unite why should not include all the types od surgery, some time easy cases have miss-conduction.  3-Bleeding was included in the complication!  4-There are some minor differences in your outcomes, but they are non-significant enough to support your conclusion.  5-How you reached the conclusion: effective safety tool. 6-Who secure the anonymity of the participants? 7-Both DM 1 and 2 are more frequent and significant. Do you think this may affect the outcomes? 8-Sepsis has many predisposing factors: Do you think it has been reduced by ISBAR only?  9- Reference 7 and 17need correction. | Thank you for wor questions and taking time for our work   1- Because of the number of surgeries we had at these time. This kind of surgery is not a surgery that we operated every day because of the complications of the surgery, the surgeon who does the surgery must be trained and in this unit at this time there was just 2 surgeons that did it. So it is because the number of patients in this period cannot be bigger. I appreciate your words and I will take the suggestions for futures investigations we are doing at this time. 2- We observed ther was no meaning with other procedures because the hospital staying and complications were very low. 3- Bleeding is one complication these kind og surgery has. 4- The conclusion maybe are non-significant but it is working nowadays to demostrate that a good communication between health providers can bring a better quality to patient and decrease the adverse events giving safety to them. 5- We think this is a safety tool because the severe events detected were reduced completely after implementing ISBAR. (Table 5 page 14) 6- Data Protection Act/law 7- Diabetes can increase your risk of problems during or after your surgery, such as: Infection after surgery (especially at the surgery site) Slower healing. Fluid, electrolyte and kidney problem. 8- Not just for ISBAR, the implementation of isbar for example it is to choose the correct profilaxis antibiotics before the surgery 9- It has been modified. Thank you. |
